# The CTCF insulator protein forms an unusual DNA structure

**DOI:** 10.1186/1471-2199-11-101

**Published:** 2010-12-21

**Authors:** Melissa J MacPherson, Paul D Sadowski

**Affiliations:** 1 Department of Molecular Genetics, University of Toronto, 1 King's College Circle, Medical Sciences Building Room 4284, Toronto, Ontario, Canada, M5 S 1A8

## Abstract

**Background:**

The CTCF insulator protein is a highly conserved zinc finger protein that has been implicated in many aspects of gene regulation and nuclear organization. The protein has been hypothesized to organize the human genome by forming DNA loops.

**Results:**

In this paper, we report biochemical evidence to support the role for CTCF in forming DNA loops. We have measured DNA bending by CTCF at the chicken HS4 β-globin FII insulator element *in vitro *and have observed a unique DNA structure with aberrant electrophoretic mobility which we believe to be a DNA loop. CTCF is able to form this unusual DNA structure at two other binding sites: the *c-myc *P2 promoter and the chicken F1 lysozyme gene silencer. We also demonstrate that the length though not the sequence of the DNA downstream of the binding site is important for the ability of CTCF to form this unusual DNA structure. We hypothesize that a single CTCF protein molecule is able to act as a "looper" possibly through the use of several of its zinc fingers.

**Conclusions:**

CTCF is able to form an unusual DNA structure through the zinc finger domain of the protein. This unusual DNA structure is formed in a directional manner by the CTCF protein. The findings described in this paper suggest mechanisms by which CTCF is able to form DNA loops, organize the mammalian genome and function as an insulator protein.

## Background

The CTCF protein, formerly known as NeP1, is an eleven zinc finger protein that is highly conserved from fruit flies to man. The protein was first identified in the chicken as a negative regulator of the c*-myc *oncogene [1] and the lysozyme gene [2]. The CTCF protein has a central zinc finger domain that shows 100% amino acid conservation between the chicken form and the human form of the protein. This central zinc finger domain is flanked by an NH_2_-terminal domain and a carboxy-terminal domain, both of which have an unknown structure. The CTCF protein has the ability to bind to different CTCF consensus sites by using different combinations of its eleven zinc fingers and is therefore frequently described as a multivalent protein [3]. These binding studies were performed by deleting different CTCF zinc fingers and observing the effects the deletions had on the ability of the protein to bind to different consensus sites [4-8]. A more recent study has determined that CTCF uses 4 to 5 core zinc fingers to bind to CTCF consensus sites [9]. Recent whole genome analyses of the CTCF binding sites in *Drosophila *and human cell lines support the idea that CTCF protein binds to a single consensus sequence [10,11].

The molecular mechanisms regulating the many diverse functions of CTCF are in part governed by the posttranslational modification of the protein. Phosphorylation of CTCF has been shown to relieve its repressive activity at the *c-myc *P2 promoter [12,13] and poly-(ADP)-ribosylation has been implicated in its role as an insulator protein [14]. In addition, we have recently shown that the posttranslational modification of CTCF by the small ubiquitin-like modifier proteins (SUMOs) contributes to its role as a transcriptional repressor at the *c-myc *P2 promoter [15].

CTCF is implicated in a diverse number of biological roles including gene repression, gene activation, chromatin insulator function, X-chromosome inactivation and the maintenance of genomic imprinting [3,16]. Recently, CTCF has been found to play a role in the organization of the mammalian genome and has been implicated in the genomic organization of the β-globin locus [17,18], the H19/Igf2 imprinting control region [19-23], the major histocompatibility complex class II genes [24] and the cystic fibrosis transmembrane conductance regulator gene locus [25]. The CTCF protein binds to approximately 15 000 sites in the human genome [11,26-29] and is hypothesized to organize the genome by forming DNA loops [30,31]. The evidence of CTCF's ability to loop DNA is the result of *in vivo *chromatin conformation capture assays (3C), and chromatin immunoprecipitation. The CTCF DNA binding site has been shown to be necessary for long-range chromatin interactions at the H19/IGF2 imprinting control region [23] and the knockdown of CTCF protein in chicken cells disrupts long-range chromatin interactions at the β-globin locus [17]. It has also been suggested that CTCF forms loops in DNA by tethering DNA to the nucleolus through its interaction with the protein nucleophosmin [31].

Since CTCF has previously been shown to bend DNA [32], we asked whether its SUMOylation altered its bending ability. In the course of answering this question, we obtained some unexpected results. Although SUMOylation had no effect on CTCF's ability to bend DNA, we found that the CTCF protein does not act as a typical DNA bending protein. The CTCF protein forms an unusual structure in DNA that we believe to be a DNA loop. This unusual DNA structure forms at all three CTCF binding sites tested: the chicken β-globin FII insulator, the chicken lysozyme gene F1 silencer element and the human *c-myc *P2 promoter. We find that the SUMOylation of CTCF does not affect its ability to form this unusual DNA structure. We discuss the possible mechanisms of DNA looping by CTCF and their roles in genome organization.

## Results

### The CTCF insulator protein forms an unusual directional DNA structure

**Figure 1:**
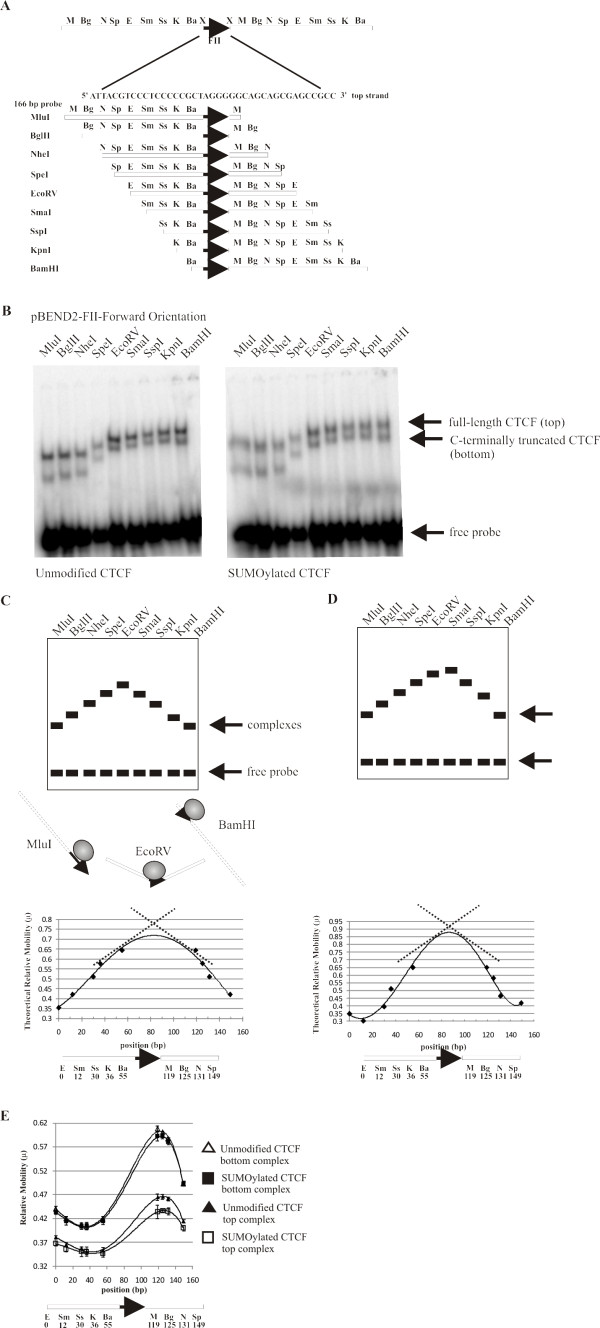
**CTCF forms unusual DNA structure at β-globin FII insulator element**. (A) Diagram of pBEND2 vector containing the FII insulator in "forward" orientation (rightward-pointing arrowhead). Restriction digestion generates 166 bp probes with FII site permuted from right (M) to left end of fragment (Ba). (B) CTCF, + or -SUMO1, was incubated with ^32^P-labelled probes. The CTCF translation product contains mixture of both full-length and C-terminally truncated protein; each exhibits same mobility pattern. (C) Expected behaviour of fragments in DNA bending assay. Mobility of protein-DNA complex decreases as DNA binding site is permuted towards centre of probe and increases as site is permuted towards ends of probe. Below schematic of electrophoretic mobilities of permuted fragments is a diagram of the best fit curve of relative mobilities (μ) as function of position (bp) from left EcoRV site to enzyme site used to generate the probe. Dotted lines indicate that bend centre is at position 80 bp. (D) Schematic of an asymmetrical DNA bend. The electrophoretic mobilities of permuted fragments plotted as in (C). The shape of best fit curve resembles that for a symmetrical DNA bend except bend centre is 90 bp. (E) Relative mobilities of CTCF-probe complexes plotted and fitted as described above. Note unusual shape of the curve compared to that in Figure 1C. SUMOylated and unmodified full length CTCF (top complex, bottom two curves) are different indicating efficient SUMOylation of CTCF *in vitro*. Top two curves (representing the bottom complexes) are similar.

CTCF has been found previously to bend DNA [32]. We initially wished to determine whether SUMOylation affected CTCF's ability to bend DNA. Therefore, we cloned the well characterized chicken β-globin FII insulator site into the XbaI site of the pBEND2 vector [33]. In this plasmid, the XbaI site is flanked by a set of tandemly repeated restriction enzyme sites (See Figure 1A). When the plasmid is digested with each of these restriction enzymes, a set of probes of equal length is generated; the DNA binding site is permuted along the length of the probe. The probes were radiolabeled with ^32^P and were incubated with CTCF that we synthesized *in vitro *[15]. The DNA-protein complexes were analysed on 4% native acrylamide gels.

In a typical DNA bending experiment, the DNA-protein complex has the slowest mobility when the DNA binding site is centrally located. Conversely, the fastest mobility occurs when the DNA binding site is located near either end of the probe. Therefore, we expected that the complexes formed by both the MluI probe and the BamHI probe would migrate more quickly than the EcoRV fragment containing the CTCF-binding site in the middle of the fragment (See Figure 1C). We tested the ability of CTCF to bend DNA using the well-characterized chicken HS4 β-globin FII insulator element. As expected, when the FII site was located near the right end of the probe (digestion with MluI), the complex migrated more quickly in the native gel than when the site was in the middle of the fragment (see Figure 1B, left). As the FII site was permuted to a more central location (digestion with BglII, NheI, SpeI and EcoRV), the mobility of the CTCF-probe complex decreased, as predicted. However, we expected that the probes generated by digestion with SmaI, SspI, KpnI and BamHI would yield complexes that would migrate progressively more quickly through the gel, mirroring the permuted probes on the opposite side of the EcoRV site as is usual in DNA bending assays. Instead, we saw even more slowly migrating complexes. We wondered if these results could be explained by CTCF binding to a second site in the pBEND2 probe itself. Therefore, we generated a probe that does not contain the FII CTCF binding site by digesting the parent pBEND2 plasmid with the enzyme BamHI. We observed no CTCF-DNA complexes with this substrate, indicating that CTCF does not bind DNA from the parent pBEND2 plasmid in the absence of the FII insulator site (data not shown).

Our original interest was to determine whether the SUMOylation of CTCF affected its ability to bend DNA; therefore, we SUMOylated CTCF quantitatively *in vitro *with SUMO1 [15]. When SUMOylated CTCF was used in the EMSA assay, the DNA bending pattern was identical to that seen using unmodified CTCF (see Figure 1B, right). Hence, the modification of the CTCF protein by SUMO did not affect its ability to deform DNA containing a CTCF binding site. The relative electrophoretic mobility (μ) of a CTCF-probe complex was calculated as the mobility of the complex divided by the mobility of the free probe. The relative mobilities of the complexes were plotted as a function of the position (bp) from the middle of the left EcoRV site to the middle of the restriction enzyme used to generate the probe (Figure 1E). The graphs were fitted with the best fit polynomial curve using Microsoft Excel. Two CTCF DNA complexes were observed in these experiments. The top complex on the electrophoretic mobility gels corresponds to the probe bound by full length CTCF, whereas the bottom complex on the gels corresponds to the probe bound by a C-terminally truncated form of CTCF caused by premature termination during *in vitro *translation. The major SUMOylation site in CTCF is found in the C-terminal domain of the protein [15]. The polynomial curves fitting the relative mobility of the top complexes of SUMOylated and unmodified CTCF are distinct, thus indicating that CTCF was efficiently SUMOylated due to the slower migration of the SUMOylated complexes (Figure 1E, bottom two curves). Conversely, when the protein is C-terminally truncated the bottom complexes exhibit similar relative electrophoretic mobilities since the C-terminally truncated CTCF is not being efficiently SUMOylated (Figure 1E, top two curves). The abnormal electrophoretic mobility exhibited by CTCF during circular permutation creates an unusual polynomial curve. Since we cannot extrapolate to the position at which the curve reaches a maximum relative mobility it was difficult to determine the centre of the CTCF-induced bend (see Figure 1C bottom). Likewise, we were unable to determine the possible differences in the bend angles induced by unmodified and SUMOylated forms of CTCF. The CTCF-induced bend is not a typical asymmetrical DNA bend (see Figure 1D). We refer to the DNA structure formed upon CTCF binding to the β-globin FII insulator element as an unusual DNA structure.

**Figure 2:**
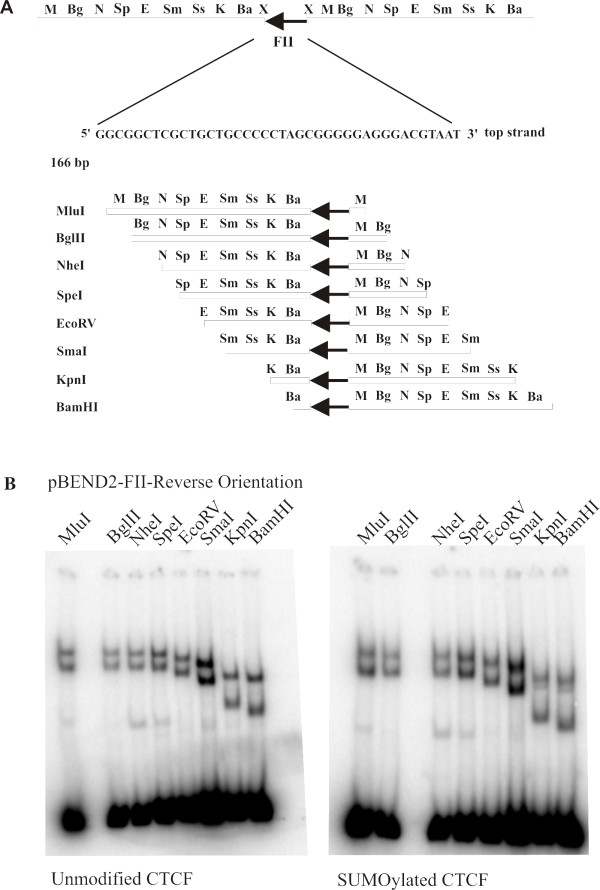
**The unusual DNA structure formed by CTCF at the β-globin FII insulator element is directional**. (A) Schematic diagram of the DNA bending vector pBEND2 containing the FII insulator element in the reverse orientation, as indicated by the leftward-pointing arrowhead. The FII site is inserted between two XbaI sites (X). Digestion of the vector with MluI (M), BglII (Bg), NheI (N), SpeI (Sp), EcoRV (E), SmaI (Sm), KpnI (K) and BamHI (Ba) results in the generation of 166 bp probes. The FII site is permuted from the right (M) to the left end of the fragment (Ba), as indicated below the sequence. Each probe contains the FII element at a different position in the reverse orientation. (B) The assays were done as in Figure 1B using ^32^P-labelled, permuted probes generated from the vector pBEND2-FII-Reverse Orientation. Note that the mobilities of the CTCF-DNA complexes are the mirror image of those observed with the pBEND2-FII-Forward vector (Figure 1B).

We then asked whether the behaviour of the CTCF-DNA complexes would persist if we inverted the FII CTCF binding site in the pBEND2 vector. We therefore generated a new plasmid called pBEND2-FII-reverse and repeated the circular permutation experiments using the new set of probes (see Figure 2A). The experiments yielded results that mirrored those of the FII forward oriented probes (see Figure 2B, left). The probe generated with BamHI formed a CTCF-DNA complex that migrated most rapidly, whereas probes generated with EcoRV, SpeI, NheI, BglII and MluI formed CTCF-DNA complexes that migrated more slowly through the gel. These results show that the altered DNA structure formed by CTCF is dependent on the orientation of the CTCF FII binding site in the pBEND2 vector. Furthermore, they show that the mobility of the CTCF-probe complex is inversely correlated with the length of the DNA downstream of the FII site. This suggests that in addition to its primary site of binding in the FII sequence, CTCF uses DNA sequence outside the FII consensus sequence to form an altered DNA structure. Once again, the SUMOylation of CTCF had no influence on the formation of the altered DNA structure (see Figure 2B, right).

Although there are several explanations for the altered DNA structure, we believe that the unusual DNA structure formed by CTCF in our bending experiments is a small DNA loop. Some of the alternative models are dealt with in the Discussion.

### The CTCF insulator protein forms a directional unusual DNA structure at two other CTCF binding sites

**Figure 3:**
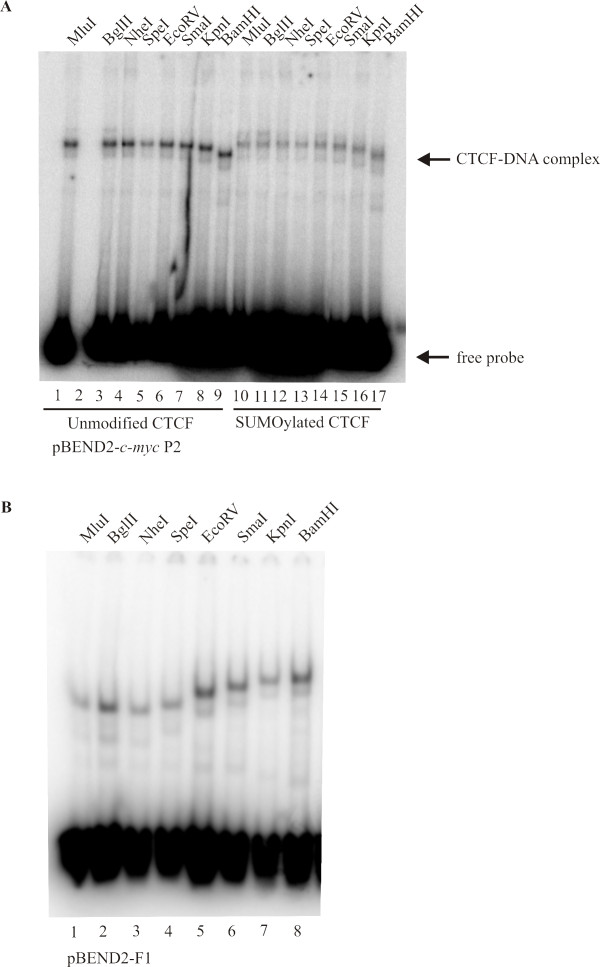
**CTCF forms the unusual DNA structure at the *c-myc *P2 promoter and at the chicken F1 lysozyme gene silencing element**. (A) The vector pBEND2-*c-myc *P2 was digested with MluI (M), BglII (Bg), NheI (N), SpeI (Sp), EcoRV (E), SmaI (Sm), KpnI (K) and BamHI (Ba) to make a series of permuted 292 bp probes The *c-myc *P2 promoter region was inserted into two XbaI sites (X). The ^32^P-labelled, permuted probes were incubated with SUMOylated CTCF lanes 10-17 and unmodified CTCF lanes 1, 3-9 (lane 2 is empty). The reactions were carried out as in Figure 1B. (B) The vector pBEND2-F1, containing the chicken lysozyme gene F1 silencing element inserted into two XbaI sites, was digested with MluI (M), BglII (Bg), NheI (N), SpeI (Sp), EcoRV (E), SmaI (Sm), KpnI (K) and BamHI (Ba) to make permuted ^32^P-labelled 178 bp probes. CTCF was made by *in vitro *transcription/translation and used directly in electrophoretic mobility shift assays with the permuted probes.

The CTCF protein binds to a rather loose consensus sequence that occurs some 15 000 times in the human genome. We were therefore interested to know whether other well-characterized CTCF binding sites would exhibit this same behaviour when placed in the pBEND2 vector. When we cloned the *c-myc *P2 promoter-binding site and the chicken lysozyme gene F1 silencer site into pBEND2, the permuted fragments also showed a similar unusual conformational behaviour in DNA bending permutation assays (Figure 3). The chicken F1 silencer site was the one previously used to characterize DNA bending by CTCF [32]. We conclude that the unusual conformation of the protein-DNA complexes occurs independently of the sequence of the CTCF DNA binding site. Incidentally, we again showed that this altered conformation occurs independently of the SUMOylation of CTCF (Figure 3A, lanes 10-17). Note that the differences in the mobilities of the DNA-CTCF complexes at the *c-myc *P2 promoter are not as striking as those seen at the F1 and FII sites since the *c-myc *P2 probes are larger than those of the F1 and FII elements. Since the mobility of the DNA-protein complexes depends upon the molecular mass of both the DNA probe and the bound protein as well as the extent of the bending, it is not unusual to see a smaller effect using a larger DNA probe.

### CTCF-DNA complexes do not involve intermolecular interactions between DNA molecules

**Figure 4:**
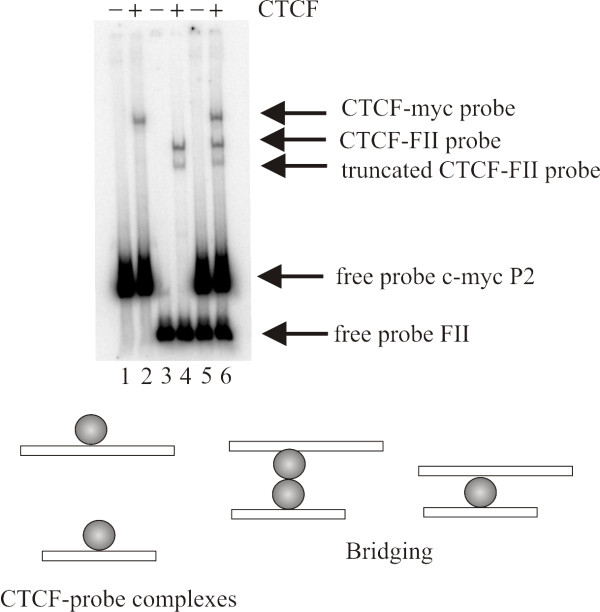
**CTCF does not bridge two DNA molecules**. CTCF was incubated with the EcoRV digested pBEND2-FII-reverse probe and/or EcoRV digested pBEND2-*c-myc*-P2 probe and electrophoretic mobility shift assays were performed. When both probes are incubated with CTCF protein (lane 6) three distinct complexes are formed; the slowest migrating protein-DNA complex is the CTCF-*c-myc*-P2 complex, the complex with an intermediate mobility is composed of the full length CTCF bound to FII probe and the complex with the fastest mobility is the C-terminally truncated CTCF protein bound to FII probe. Significantly, no higher molecular weight complexes indicative of dimeric complexes of each probe bridged by CTCF are seen. A schematic of the possible bridged structures is illustrated under the gel. A bridged complex can be formed between one or two bound CTCF molecules (spheres).

The slowly migrating complexes seen in the bending assays might have arisen through the multimerization of two DNA fragments mediated by CTCF. To address this question, we performed a mixing experiment using separate probes containing either the *c-myc *P2 promoter region or the FII insulator. These were prepared by radiolabeling EcoRV digested pBEND2-*c-myc *P2 and pBEND2-FII-reverse plasmids, respectively. The FII probe is smaller than the *c-myc *P2 fragment and the two probes are easily resolved on a 4% native polyacrylamide gel (see Figure 4, lanes 1 and 3). Upon the addition of CTCF, the CTCF-*c-myc *P2 complex and the CTCF-FII complexes are also easily resolved on the native gel (Figure 4, lanes 2 and 4). If CTCF were acting as a DNA bridging protein between the two probes, then the incubation of CTCF with both probes should cause the appearance of an additional higher molecular weight complex migrating behind the *c-myc *P2-CTCF complex (see schematic below the gel). As seen in lane 6 of Figure 4, no such complex is detected. Therefore, we conclude that CTCF is not forming an intermolecular bridge between two DNA molecules in our experiments.

### The zinc finger domain of CTCF is sufficient for the formation of the unusual DNA structure

**Figure 5:**
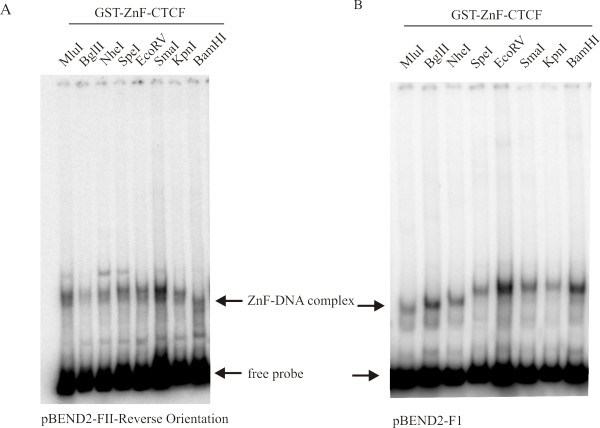
**The CTCF zinc finger domain is responsible for the formation of the unusual DNA structure**. (A) Electrophoretic mobility shift assays were performed using the purified zinc finger domain and radiolabeled permuted probes containing the β-globin chicken FII insulator element in the reverse orientation. The zinc finger domain exhibits the similar mobility pattern when bound to the FII probes, as does the full length CTCF (see Figure 2B). The extraneous bands running behind the CTCF-DNA complexes in the MluI, NheI, and SpeI lanes are present in the probes in the absence of added CTCF and are of unknown origin. (B) The experiment was repeated with the radiolabeled permuted probes containing the chicken F1 lysozyme silencer element. Once again, the zinc finger domain is sufficient for the formation of the unusual DNA structure. Note that the complexes of CTCF with probes generated by digest with MluI, BglII and NheI show an increased mobility at the F1 element while the remaining probe-CTCF complexes show a decreased electrophoretic mobility in the native gel.

The CTCF protein is thought to use multiple permutations of its zinc fingers to bind to its highly diverse DNA binding sequences [3,4,6-8]. Therefore, it was of interest to learn whether the ability to form the altered DNA structure on the pBEND2 vectors also resided in the zinc finger domain. We used permuted probes from the pBEND2-FII-reverse plasmid in electrophoretic mobility shift assays with the CTCF zinc finger domain (Figure 5A). When the zinc finger domain is incubated with the pBEND2-FII-reverse BamHI fragment (the FII binding site at the end of the probe), the DNA-protein complex migrates rapidly through the acrylamide gel. When the probe was prepared by digesting with MluI, (FII binding site is at the opposite end of the probe), a slower migrating DNA-protein complex was obtained. Therefore, the zinc finger domain of CTCF is sufficient for the formation of the unusual DNA structure at the FII insulator element. We obtained the same circular permutation results using permuted probes containing the chicken F1 lysozyme gene silencer (Figure 5B). Because of its reduced molecular mass, the zinc finger domain produces a smaller effect on electrophoretic mobility than that of the full-length protein during circular permutation assays. When the results of the circular permutation assay using the CTCF zinc finger domain are plotted, the curve is not indicative of an asymmetric bend but is once again unique (data not shown). As can be seen, three of these probes (MluI, BglII and NheI) show the increased mobility at the F1 element. We conclude that CTCF's ability to form the unusual DNA structure resides in its zinc finger domain.

### The formation of the unusual DNA structure by CTCF is not dependent on a specific DNA sequence downstream of the CTCF binding site

**Figure 6:**
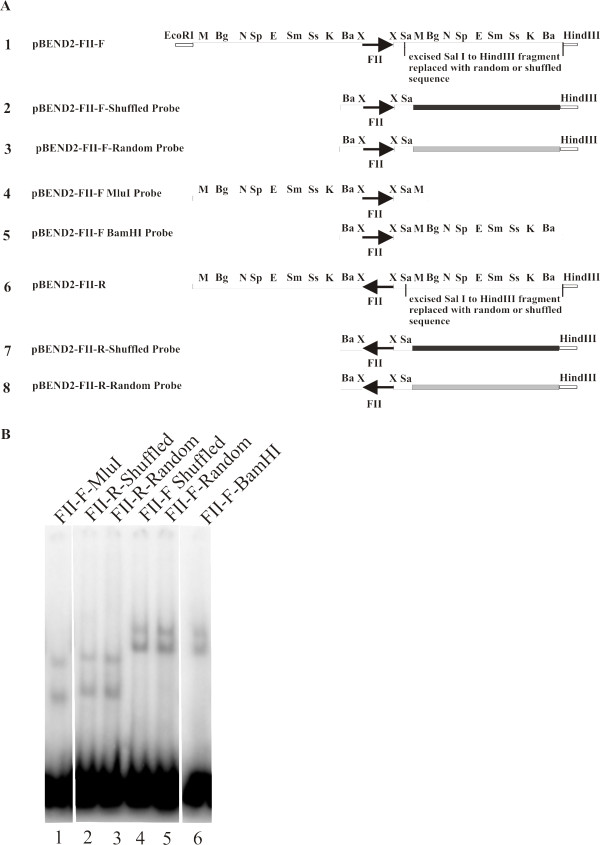
**The formation of unusual DNA structure by CTCF is not dependent on DNA sequence composition downstream of CTCF binding site**. (A) Construction of probes: the pBEND2-FII-forward or reverse plasmids (lines 1 and 6) were digested with HindIII and SalI to remove DNA sequence to the right of the FII insulator element. This sequence was replaced with a shuffled version of the original sequence (black line, lines 2 and 7) or random DNA sequence (grey line, lines 3 and 8). Lines 4 and 5 show control probes containing original FII site and downstream DNA sequence digested with MluI or BamHI. (B) Mobility shift assays were performed by incubating CTCF with radiolabeled 173 bp probes generated by HindIII and BamHI double restriction digests of pBEND2-FII-reverse-random, pBEND2-FII-reverse-shuffled, pBEND2-FII-forward-random or pBEND2-FII-forward-shuffled. In lane 1, the control MluI digested pBEND2-FII-Forward probe shows the rapidly migrating CTCF-probe complexes. Lanes 2 and 3 show the FII-reverse shuffled and random probe complexes have a similar mobility to the control MluI complexes in lane 1. Lanes 4 and 5 show that the FII-forward shuffled and random probe complexes have a similar mobility to the control BamHI probe in lane 6. Therefore, DNA sequence downstream of FII binding site does not affect CTCF's ability of to form a DNA loop. All samples were run on the same polyacrylamide gel, although the lanes were re-arranged electronically.

We have observed that when the length of the DNA "downstream" of the FII site increased, the mobility of the CTCF-DNA complex in the native gel decreased. This suggests that CTCF binds to its consensus-binding site in a unique orientation and uses DNA downstream of the FII consensus sequence to form the unusual DNA structure with reduced electrophoretic mobility. To determine whether the actual DNA sequence downstream of the FII consensus affected the ability of CTCF to form the unusual DNA structure, we replaced the DNA sequence downstream of the FII site with two new DNA sequences. In the plasmid pBEND2-FII-Forward-Shuffled we replaced the original sequence with a shuffled version of the original downstream sequence. We also constructed the plasmid pBEND2-FII-Forward-Random by replacing the original downstream sequence with a random DNA sequence composed of a 50% A+T and 50% G+C base composition (Figure 6A). When probes were prepared by digesting the new plasmids with BamHI and HindIII we observed that CTCF is still able to form the unusual DNA structure regardless of the DNA sequence downstream of the FII binding site (Figure 6B, lanes 4, 5 versus lane 6). As controls, we also cloned both the shuffled DNA sequence and the random DNA sequence into the pBEND2-FII-Reverse plasmid. When the same probes were prepared with the control clones, CTCF did not form the DNA structure showing that the reduced mobility of the experimental probes was not due to the new DNA sequences cloned downstream of the FII site (Figure 6B, lanes 2, 3 versus lane 1).

### CTCF phases DNA in a manner dependent upon the orientation of the CTCF binding site

**Figure 7:**
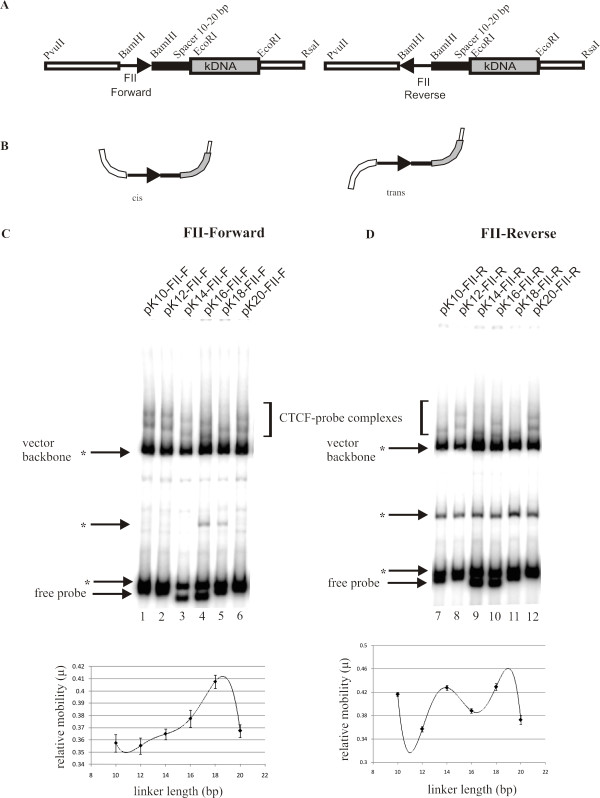
**CTCF exhibits orientation dependent directed bend at FII insulator element**. (A) κ-DNA contains phased A rich tracts with an intrinsic bend toward the minor groove. FII insulator element points towards κ-DNA (forward) or away from it (reverse). Helical phasing between sites is varied by increasing length of DNA spacer (black box) by 2 bp increments (10 to 20 bp) over one helical turn of the DNA. Plasmids digested with RsaI and PvuII gave 391bp to 401bp probes containing FII site. NheI was included to cleave a plasmid backbone fragment. (marked with asterisk, does not bind CTCF). (B) Schematic diagram of *cis *and *trans *isomers. C*is *isomer migrates more slowly than *trans *isomer. (C) Phasing experiment: FII site in forward orientation. Two CTCF DNA complexes are seen. Lower complex, formed by truncated CTCF, is obscured by vector backbone fragment (*, lane 5). Complex migrates most slowly when the protein-induced and sequence-directed bends are additive ("*cis*" isoform, lanes 1 and 6). When bends are out of phase ("*trans*" isoform), complex has greatest mobility (lanes 4 and 5). Relative mobilities of protein-DNA complexes normalized to those of free probes are plotted relative to spacer length. Best fit polynomial curve was determined using Microsoft Excel. (D) Phasing experiment: FII site in reverse orientation. Two CTCF DNA complexes are seen. Lower complex is obscured by contaminating radiolabeled vector fragment (7, 9 and 11). "*Cis*" isomer and "*trans*" isomers are formed in alternating manner when spacer length increases by 2 bp.

The DNA bending assays using the pBEND2 vector cannot distinguish between a directional bend in the DNA and DNA flexure. DNA phasing assays indicate whether the bend has directionality as opposed to a random DNA flexure in which the DNA may be bent in any direction. We used the phasing vectors of Zinkel *et al*. [34] to determine the behaviour of the CTCF-induced bend. These vectors contain a BamHI cloning site for the DNA-binding site of interest (in this case the FII site) joined to a kinetoplast DNA sequence. The kinetoplast DNA consists of sequences of A-tract repeats that cause a sequence-directed bend toward the minor groove of the helix. The length of the linkers between the two sites is varied over 1 turn of the DNA helix: 10, 12, 14, 16, 18 and 20 bp. As can be seen in Figure 7A, the FII insulator element was cloned into the BamHI site of the phasing vectors in two orientations. The forward orientation occurs when the FII site is pointing towards the kinetoplast DNA (κDNA) and the reverse orientation occurs when the FII site is pointing away from the κDNA as indicated by an arrow. When the FII site is cloned "facing" the kinetoplast DNA (forward orientation), CTCF induces a typical DNA bend rather than a DNA flexure. The migration of the DNA-probe complexes changes depending on the orientation of the sequence-induced bend of the kinetoplast DNA and the CTCF induced bend in the FII insulator element. The minimal migration of the CTCF-DNA complexes corresponds to the "cis" isomer, the DNA conformation where the protein-induced bend and the sequence induced bends are in the same direction as shown by the schematic in Figure 7B. When these bends are in the opposite direction, the CTCF-DNA complexes show maximal migration in the polyacrylamide gel; this corresponds to the "trans" isomer (Figure 7B). In our phasing experiments, when the FII site is in the forward orientation the CTCF-DNA complexes exhibit minimum mobility at a linker length of between 10 bp and 12 bp (Figure 7C, "cis" configuration) whereas maximum mobility of the CTCF-probe complex is seen when the linker length is approximately 18 bp ("trans" configuration). The relative mobilities of the top CTCF-DNA complexes (full length CTCF) normalized to the mobility of each free probe are plotted below the gel in Figure 7C. The best fit polynomial equation was fitted to the data using Microsoft Excel to determine the linker length corresponding with both maximal and minimal CTCF-DNA complex mobilities. The CTCF protein is phasing DNA over a length of 10 bp, which corresponds to one helical repeat as seen by the overlap between the mobilities of the CTCF-probe complexes at linker lengths of 10 bp and 20 bp in the graph. This experiment confirms that when the FII site is cloned "facing" the kinetoplast DNA (forward orientation), CTCF induces a typical DNA bend rather than a DNA flexure (Figure 7C). However, when the orientation of the FII site was reversed we observed an unusual electrophoretic behaviour of the CTCF-DNA complex. The mobility pattern is multiphasic, seemingly varying with a periodicity of 2 bp (Figure 7D). Since our circular permutation assays yielded these unusual results we were unable to calculate the centre of the DNA bend and are therefore unable to determine the direction of the bend using phasing analysis.

We believe that the complex multiphasic mobility pattern is due to a CTCF-induced directional bend towards the kinetoplast DNA in addition to an unusual DNA structure formed towards the PvuII restriction enzyme site. These complex DNA conformations may not phase because the unusual DNA structure is not a fixed structure. When the phasing analysis was done in the forward orientation, the unusual DNA structure may have been unable to form due to the topological constraints posed by the bent kinetoplast DNA. Therefore, the CTCF-induced bend alone is responsible for the DNA phasing.

## Discussion

In this paper we have presented biochemical evidence that shows CTCF is able to form an unusual DNA structure. We believe that this DNA structure is a CTCF-induced DNA loop and that CTCF acts as a DNA looping protein. We show that this unusual DNA structure is formed at the chicken β-globin FII insulator element and that its formation depends upon the orientation of the FII site. The unusual DNA structure also forms at two other well-characterized CTCF binding sites: the *c-myc *P2 promoter and the chicken lysozyme gene F1 silencer element. These results were unexpected since Arnold *et al*. [32] had previously characterized CTCF (formerly named NeP1) as a DNA bending protein. However, there are several differences between our circular permutation assay and those of these authors. We examined the effect of CTCF protein only on a circular permutation substrate that contained a single CTCF binding consensus sequence. Arnold *et al*. [32] examined a complex protein mixture of CTCF (NeP1), TR, and RXR in their circular permutation assays. The large number of protein-DNA complexes formed in their mobility shift assays may have obscured the aberrant electrophoretic mobilities of the CTCF-probe complexes alone. Careful re-examination of their data shows it to be consistent with our experiments.

**Figure 8:**
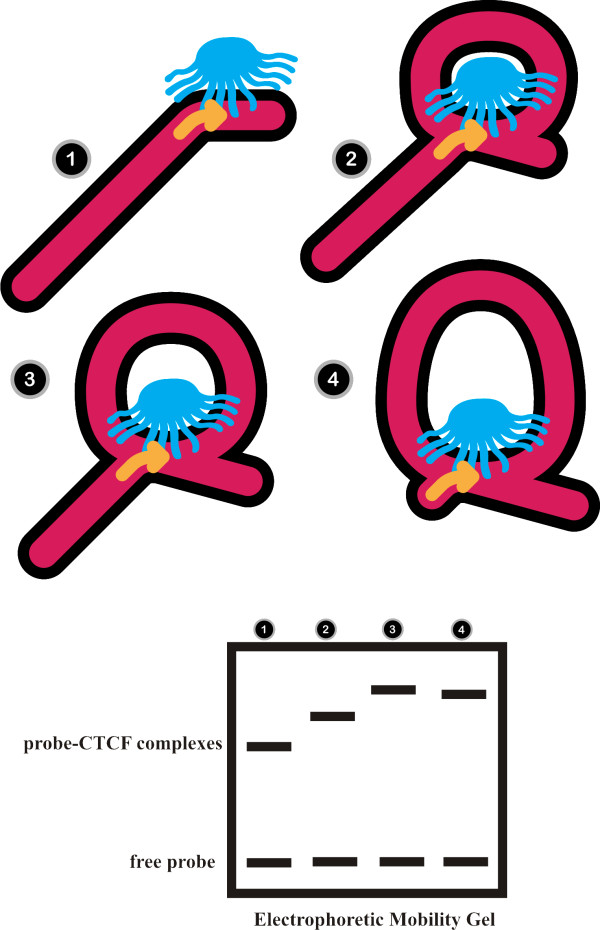
**A speculative model of the CTCF-probe topologies in the DNA permutation experiments**. A schematic model of the CTCF looping process. CTCF is shown in blue with its eleven zinc fingers as protrusions. The protein uses a set of core zinc fingers to bind to the CTCF binding site (indicated by an arrow), leaving the remaining zinc fingers available for DNA loop formation. In 1, CTCF binds to the CTCF binding site near the end of the probe causing a bend in the DNA (shown in pink). As the length of the probe downstream of the FII insulator element increases, the length of the available DNA for DNA loop formation increases and therefore the size of the DNA loop also increases, as shown in 2 and 3. This results in a decreased electrophoretic mobility (bottom, lanes 1 through 3). As the length of the probe upstream of the FII insulator element decreases to its minimum length and the size of the loop is at its greatest (as shown in 4), the mobility of the CTCF-probe complex increases slightly in the native acrylamide gel (bottom, lane 4). This is due to the reduction in "drag" caused by the DNA upstream of the FII site as the CTCF-probe complex migrates through the acrylamide gel. No attempt is made to portray the sign (+ or -) of the crossing nodes.

We believe that the unusual DNA structure formed by CTCF in the circular permutation assays is a small DNA loop and we have depicted the results of our circular permutation experiments and the CTCF loop topologies in a speculative schematic diagram in Figure 8. As indicated in our model, as the length of the probe downstream of the CTCF binding site increases a DNA loop is formed (Figure 8, lanes 1 and 2) and the electrophoretic mobility of the CTCF-DNA complex is decreased. As the length of the probe upstream of the FII insulator element decreases and the length of the probe downstream of the binding site increases, the size of the loop is also increased (Figure 8, lane 3). In lane 4, the electrophoretic mobility of the CTCF-DNA complex is slightly greater than the mobility exhibited by the complex in lane 3. We believe that this is due to the reduction in "drag" caused by the DNA upstream of the FII site as the loop reaches its maximal size.

There are several alternative models that could account for the unusual DNA topology caused by CTCF. One possible explanation might be that CTCF induces formation of single-stranded regions of DNA downstream of the FII binding site. However, when we probed the altered DNA structure with potassium permanganate or diethyl pyrocarbonate, the presence of single stranded DNA was not detected by DNA footprinting (not shown). Therefore, we conclude that the altered DNA structure is therefore not caused by melting of the DNA.

A second explanation is that the FII binding site nucleates the binding of multiple CTCF molecules downstream of the FII binding site. For example, multiple RecA protein molecules nucleate onto single stranded DNA in a unidirectional manner [35]. Interestingly, a C-terminally truncated form of CTCF is present in the *in vitro *translation reaction. This complex was identified using "supershift" experiments in the presence of a C-terminal-specific antibody (data not shown). If the complexes formed at the FII binding site are caused by CTCF nucleation, we might have expected to detect a mixed protein-DNA complex containing both the truncated and full-length CTCF. Since we do not detect this mixed complex, we conclude that the CTCF-DNA complexes contain a single molecule of CTCF or its C-terminal truncation.

It is possible that the nucleation of other proteins present in the reticulocyte lysate accounts for the aberrant mobility of the CTCF-probe complexes. However, we note that the purified GST-zinc finger domain of CTCF exhibits the same overall electrophoretic mobility pattern as the *in vitro *translated CTCF lysates. The presence of such proteins in the GST-zinc finger protein purified from *E. coli *is unlikely.

A further possible explanation for the aberrant DNA structure is the wrapping of DNA around CTCF. Circular permutation experiments using the mitochondrial protein mtTF1, a DNA wrapping protein, show a protein-DNA mobility pattern similar to those observed in classical circular permutation experiments [35]. While these other models may provide an explanation for the abnormal DNA structure formed by CTCF, the DNA looping model is the strongest explanation and fits well with the *in vivo *data describing CTCF's role in mediating long-range chromatin interactions.

Our bending results provide insight into the molecular mechanisms of the CTCF protein. It is of interest to note that the unusual DNA structure formed by CTCF is directional. CTCF uses DNA downstream of the binding site to form the structure while it does not appear to use DNA upstream of the binding site. Interestingly, the *Drosophila *insulator protein Su (Hw), which is also a zinc finger protein that binds to the gypsy insulator element, has also been studied in circular permutation experiments. Like CTCF, Su(Hw) protein is believed to contain twelve zinc fingers in a central protein domain and like CTCF, the Su(Hw) protein bends DNA in an unusual manner [36]. These authors attributed the aberrant behaviour to either an increase in flexibility or a melting of the DNA by Su(Hw).

In our experiments, the zinc finger domain of CTCF is sufficient for both the bending and looping activities. Arnold *et al*. [31] used extracts from mammalian cells transfected with a CTCF zinc finger domain construct to conclude that CTCF's DNA bending ability resided outside the zinc finger domain of the protein. It is conceivable that our results differ from those of Arnold *et al*. since we used a partially purified zinc finger domain instead of a complex cell lysate. The Su(Hw) protein needs its zinc fingers to bind DNA but requires the presence of its acidic C-terminal domain for DNA bending. This suggests that the mechanism by which Su(Hw) forms an unusual DNA structure may differ from that used by CTCF.

We determined that the nucleotide sequence of the DNA outside the FII binding site is not critical for the formation of the DNA loop. We speculate that these results could provide mechanistic insight into the ability of CTCF to organize the human genome. If CTCF were to bind specifically to a core consensus sequence it might then make a DNA loop using any nucleotide sequence. The protein might conceivably be able to make a DNA loop at any of the 15 000 sites in the genome to which it has been shown to bind.

Previous studies have attributed CTCF's ability to bind to different CTCF consensus sites to its use of different combinations of its eleven zinc fingers [3]. These studies were performed by deleting different CTCF zinc fingers and observing the effects the deletions had on the ability of the protein to bind to different consensus sites [4-8]. A more recent study has determined that CTCF uses 4 to 5 core zinc fingers (zinc fingers 4-8) that are critical to providing high affinity binding to a 12bp core sequence in CTCF consensus sites [9]. Recent whole genome analyses of CTCF binding sites in *Drosophila *and human cell lines support the idea that CTCF protein binds to a single consensus sequence [10,11].

If CTCF needs only 4 to 5 zinc fingers to bind to consensus sites (Figure 8), then the remaining six or seven zinc fingers might be free to bind DNA non-specifically to form a loop. A crystal structure of CTCF in the presence of DNA would add significant insight into its ability to form a DNA loop and its combinatorial use of zinc finger during DNA binding. The analysis of CTCF's DNA binding ability by surface plasmon resonance has shown that the binding of CTCF to DNA is a two-stage reaction [7]. It is possible that the first stage of the binding reaction is due to the initial binding of CTCF to the consensus site followed by a second binding step whereby CTCF forms a DNA loop. Alternately, the initial binding may non-specific followed by binding to the consensus site.

The CTCF protein has been previously shown by other workers to act as a DNA bridging protein [36]. In electrophoretic mobility shift assays, CTCF was able to form an intermolecular complex between two probes that contained target site 3 or 4, respectively, from the mouse IGF2/H19 imprinting control region. We were unable to detect bridged complexes between probes containing the FII insulator element and the *c-myc *P2 promoter. It is possible that CTCF acts in a different manner when binding to the IGF2/H19 ICR than at other CTCF consensus sites. It is also possible that our EMSA experiments did not contain sufficient amounts of CTCF protein to overcome the necessary entropic costs of forming bridged, cross-joined or sandwiched structures. Several proteins are known to form these DNA structures; however, sufficiently high binding energy is needed to achieve these DNA topologies. The lac repressor protein is known to form such structures only when the protein is present at sufficiently high concentrations [37]. It is difficult to compare the amount of CTCF protein generated in our *in vitro *translation reaction to those obtained by Pant *et al*. for use in their gel shift experiments [37].

The experiments presented in our paper add biochemical evidence to CTCF's loop-forming ability. DNA looping is a central phenomenon in gene regulation in both prokaryotes and eukaryotes. There are several well-characterized models of DNA looping in the control of prokaryotic gene expression including the *E. coli lac *and *deo *operons as well as the integrase protein of the bacteriophage lambda [38,39]. In all of these models protein:protein interactions either through the homodimerization of looping proteins or the heterodimerization of several looping proteins are required for DNA loop formation. The mechanism of CTCF DNA loop formation that we observed seems to be somewhat different. We propose a model of CTCF insulator function whereby a single CTCF protein molecule could form a DNA loop in an orientation dependent manner instead of the dimerization of two CTCF protein molecules or a bridging of two DNA molecules mediated by CTCF. By extension, a CTCF-dependent insulator element is a *cis *regulatory element that is the site of a DNA loop and the enhancer blocking ability of a DNA insulator function could be the result of the formation of the loop. It is interesting to note that Kyrchanova *et al*. [40] recently demonstrated that functional pairing interactions between *Drosophila *insulators was an orientation dependent interaction. Their findings fit nicely with our discovery of the orientation dependence of the unusual DNA structure formed by CTCF.

The results we have presented in this paper are a starting point towards a greater understanding of CTCF's DNA looping ability and the molecular mechanisms regulating this ability.

## Conclusions

We conclude that the CTCF insulator protein is able to form an unusual DNA structure *in vitro *that we believe is a DNA loop. This unusual DNA structure is formed at several CTCF binding sites and is formed in a directional manner. The CTCF zinc finger domain is sufficient for the formation of the unusual DNA structure. CTCF uses DNA downstream of the CTCF binding site to form the unusual DNA structure but the sequence of this downstream DNA does not affect the formation of the structure. When the DNA sequence downstream of CTCF is topologically constrained, the unusual DNA structure is unable to form and CTCF acts as a DNA bending protein. The results of this study could provide mechanistic insights into CTCF's ability to mediate long-range chromatin interactions and form DNA loops.

## Methods

### Plasmids and Cloning

Oligonucleotides containing CTCF binding sequences in the chicken β-globin FII HS4 insulator element (pr2214 and pr2215) and chicken lysozyme F1 gene silencer (pr2236 and pr2237) are described in Table 1. The oligonucleotides were annealed, digested with the restriction enzyme XbaI and cloned into the XbaI site in the DNA bending vector pBEND2 [33]. Plasmids containing the FII site in both orientations were isolated. We arbitrarily name the orientation in which the FII site is pointing "towards" the HindIII site in the pBEND2 vector as the "forward" orientation. Conversely, when the FII site is pointing towards the EcoRI site in the pBEND2 vector the orientation is defined as the "reverse" orientation (See Figure 1A and 2A). A fragment containing the *c-myc*-P2 promoter was amplified by PCR using the primers pr2216 and pr2217 (Table 1) as described previously [15]. The PCR product was digested with the restriction enzyme XbaI and was cloned into the XbaI site in the vector pBEND2 resulting in the vector that was called pBEND2-*c-myc*-P2. Only plasmids containing the forward orientation were isolated.

**Table 1:** Oligonucleotides used in this study.

Primer Name	Clone	Sequence	Orientation	Description
pr2214	pBEND2-FII	ctag[tctaga]attacgtccctcccccgctagggggcagcagcgagc cgcc[tctaga]ctag	Top	[XbaI]
pr2215	pBEND2-FII	ctag[tctaga]ggcggctcgctgctgccccctagcgggggagggac gtaat[tctaga]ctag	Bottom	[XbaI]
pr2216	pBEND2-c-myc-P2	ctag[tctaga]gatcgcgctgagtataaaagc	F	[XbaI]
pr2217	pBEND2-c-myc-P2	ctag[tctaga]cctattcgctccggatctc	R	[XbaI]
pr2223	pK FII phasing vectors	cgc[ggatcc]attacgtccctcccccgctagggggcagcagcgagc cgcc[ggatcc]gcg	Top	[BamHI]
pr2224	pK FII phasing vectors	cgc[ggatcc]ggcggctcgctgctgccccctagcgggggagggac gtaat[ggatcc]gcg	Bottomn	[BamHI]
pr2236	pBEND2-F1	ctag[tctaga]aattgagacctctactggatagctatggtatttacatgt ctttttgcttag[tctaga]ctag	Top	[XbaI]
pr2237	pBEND2-F1	ctag[tctaga]ctaagcaaaaagacatgtaaataccatagctatccag tagaggtctcaatt[tctaga]ctag	Bottom	[XbaI]
pr2228	pBEND2-FII random	[tcgac]ttcctattatcgtccgaactccgaaccctctgtcttgtactgcc tggcacagcactagaggaatccctatcgttctggcatcaaccatgatt atacgctgctcggaatg[a]	Top	[SalI][HindIII]
pr2229	pBEND2-FII random	[agctt]cattccgagcagcgtataatcatggttgatgccagaacgata gggattcctctagtgctgtgccaggcagtacaagacagagggttcgg agttcggacgataataggaa[g]	Bottom	[HindIII][SalI]
pr2232	pBEND2-FII shuffled	[tcgac]gattctgacagtgagatctgtgagatttcagttcgcggatca ccgtacttgatcccaggctaagacggaaagtaaggaaacgcctgct ccagctgtaccggtccccgta[a]	Top	[SalI][HindIII]
pr2233	pBEND2-FII shuffled	[agctt]tacggggaccggtacagctggagcaggcgtttccttactttc cgtcttagcctgggatcaagtacggtgatccgcgaactgaaatctca cagatctcactgtcagaatc[g]	Bottom	[HindIII][SalI]
pr1964	pGEX4T-1-ZnF-CTCF	ttcgc[ggatcc]ggtgtaaagaaaacattccagtgt	F	[BamHI]
pr1965	pGEX4T-1-ZnF-CTCF	atccg[ctcgag]acagttatctgcatgtcttgccat	R	[XhoI]

To construct the shuffled clones, we randomized the original 115 nt DNA sequence from the SalI restriction enzyme site to the HindIII restriction enzyme site in the pBEND2 vector using the online Bioinformatics tool, the Sequence Manipulation Suite [41]. The shuffled oligonucleotides pr2232 and pr2233 (Table 1) were annealed and phosphorylated using polynucleotide kinase (New England Biolabs). The primers were engineered to contain SalI and HindIII restriction enzyme sites for ligation into the SalI and HindIII sites in the pBEND2-FII-Forward or Reverse vectors. The result was the formation of the plasmids pBEND2-FII-Forward or Reverse-Shuffled in which the tandemly repeated restriction enzyme sites to the right of the XbaI site were replaced with the shuffled sequence. The random clones were constructed in a similar manner except that a random 115 nt DNA sequence of 50% A+T and 50% G+C base composition was generated using the Sequence Manipulation Suite [41]. The random oligonucleotides pr2228 and pr2229 were cloned into pBEND2-FII-Forward or Reverse to form the plasmids pBEND2-FII-Forward or Reverse-Random as described above (see Figure 6A).

The phasing clones were constructed by excising the 156 bp BamHI-BamHI DNA fragment from the plasmids pK10, pK12, pK14, pK16, pK18 and pK20 and replacing it with annealed oligonucleotides that contained the chicken β-globin FII HS4 insulator element cut with BamHI (see Table 1 and Figure 7A). Clones in both orientations were isolated.

### Purification of the zinc finger domain of CTCF

The zinc finger domain of CTCF was amplified by PCR from a mouse CTCF cDNA clone using the primers pr1964 (BamHI) and pr1965 (XhoI) (Table 1) and was cloned into the BamHI and XhoI restriction sites in the vector pGEX4T-1 (GE Healthcare) to create an NH_2_-terminal glutathione *S*-transferase (GST) fusion protein. The GST-fusion of the zinc finger domain was purified as previously described [15]. The purity of the GST-ZnF-CTCF domain was determined to be about 37% pure by scanning a Coomassie stained SDS-PAGE using the program ImageJ.

### *In vitro *transcription/translation of CTCF and *in vitro *SUMOylation

Full length CTCF was prepared by *in vitro *transcription/translation as described previously. The full length CTCF was posttranslationally modified by SUMO 1 *in vitro *using a SUMOylation control kit purchased from LAE Biotech International (catalogue no. K007) as described with the following modifications [15]. Briefly, 2 μL of *in vitro *translated CTCF were incubated with the recommended amounts of E1 and E2 enzymes and 20 mM ATP with or without SUMO 1 protein in a 12 μL reaction mixture for one hour at 37°C. The SUMOylation of CTCF was quantitative as shown previously [15].

### Circular Permutation Assay

Probes were prepared from the pBEND2 clones by digesting plasmid DNA with the following restriction enzymes: MluI, BglII, NheI, SpeI, EcoRV, SmaI, SspI, KpnI and BamHI. The digested probes were purified from an agarose gel using the MinElute Gel Purification Kit (Qiagen) and 5' labeled with γ-^32^P-ATP (Perkin Elmer) and T4 Polynucleotide Kinase (New England Biolabs). Electrophoretic mobility shift assays were carried out as described previously with the following modifications. Briefly, 2 μL of SUMOylated or unmodified CTCF from the SUMOylation reactions were used in each gel shift reaction. In experiments where the effects of SUMOylation were not being assayed, 0.5 μL to 1 μL of the *in vitro *translate was used in each gel shift reaction. In experiments using the GST-fusion of the zinc finger domain of CTCF, 250 ng of the purified protein domain was used in each gel shift reaction. The reactions were run on 4% native polyacrylamide gels in 0.25X TBE at 9 V/cm. The gel dimensions for the FII and F1 mobility shifts with full-length CTCF were 10 cm × 7 cm, while gels 19.5 cm × 16 cm were used to analyse the *c-myc *P2 promoter and the GST-zinc finger domain mobility shifts. The dried gels were exposed to a phosphor screen and imaged using a Phosphorimager. The electrophoretic mobilities of the CTCF-FII forward and reverse probe complexes and free probes were measured using ImageQuant software. The relative electrophoretic mobility (μ) of a CTCF-probe complex was calculated as the mobility of the complex divided by the mobility of the free probe. The relative mobilities of the complexes were plotted as a function of the position (bp) from the middle of the left EcoRV site to the middle of the restriction enzyme used to generate the probe and the graphs were fitted with the best fit polynomial curve using Microsoft Excel.

### Phasing Analysis

The fragments for the phasing experiments were prepared by digesting the pK10, pK12, pK14, pK16, pK18 and pK20 plasmids containing the FII insulator element in either the forward or reverse orientation, with the restriction enzymes RsaI, PvuII and NheI. Since the probes were not gel purified, we included the NheI restriction enzyme to digest a 464 bp RsaI vector fragment that would otherwise co-migrate with the FII insulator probes. The 391 bp to 401 bp RsaI to PvuII fragment contains the FII insulator element. Fragments were 5' labeled and electrophoretic mobility shifts were performed as described above, using 19.5 cm × 16 cm 4% native PAGE gels. The electrophoretic mobilities of the CTCF-FII forward and reverse phasing probe complexes and free probes were measured using ImageQuant software. The relative electrophoretic mobility (μ) of a CTCF-probe complex was calculated as the mobility of the complex divided by the mobility of the free probe. The relative mobilities of the complexes were plotted as a function of the linker length (bp). The graphs were fitted with the best fit polynomial curve using Microsoft Excel.

## Authors' contributions

MJM designed and performed the experiments described in this paper. Both MJM and PDS conceived the study and wrote the manuscript. Both authors read and approved the final manuscript.
